# Hearing loss in congenital toxoplasmosis detected by newborn screening

**DOI:** 10.1016/S1808-8694(15)30746-1

**Published:** 2015-10-19

**Authors:** Gláucia Manzan Queiroz de Andrade, Luciana Macedo de Resende, Eugênio Marcos Andrade Goulart, Arminda Lucia Siqueira, Ricardo Wagner de Almeida Vitor, José Nelio Januario

**Affiliations:** 1 MSc. Assistant Professor - Department of Pediatrics FM-UFMG, NUPAD Researcher; 2 MSc. Assistant Professor of Speech and Hearing Therapy - FM-UFMG; 3 PhD. Adjunct Professor - Department of Pediatrics - FM-UFMG; 4 PhD. Adjunct Professor - Department of Statistics - UFMG, NUPAD Researcher; 5 PhD. Adjunct Professor - Department of Parasitology - ICB-UFMG; 6 MSc. Assistant Department of Clinical Medicine - FM-UFMG, NUPAD Researcher

**Keywords:** hearing loss, congenital toxoplasmosis, newborn screening

## Abstract

Congenital toxoplasmosis may cause sensorineural deficit in up to 20% of the patients and proper treatment in the first year improves prognosis. In Brazil, this infection's impact on hearing impairment is unknown.

**Aim:**

To evaluate hearing of newborns with congenital toxoplasmosis identified by the newborn screening service.

**Method:**

This prospective study analyzed children with congenital toxoplasmosis identified by newborn screening (IgM anti-T.gondii) in Belo Horizonte during 2003/2004. The presence of IgM and/or IgA in the first 6 months or IgG at 12 months-of age in serology was used as case definition. Hearing tests were carried out at the time of diagnosis and 12 months later, including behavioral audiometry, evoked otoacoustic emission and brainstem evoked responses audiometry.

**Results:**

Among 30.808 screened children (97% of live births), 20 had congenital toxoplasmosis, 15 (75%) were asymptomatic at birth. Nineteen children were evaluated by hearing tests. Four had sensorineural impairment (21.1%). One child had other risk factors for hearing impairment; the other three had no other risk factors but toxoplasmosis. Two properly children treated still had hearing loss, in disagreement with current literature.

**Conclusion:**

Results suggest that congenital toxoplasmosis, common in Brazil, is a risk factor for hearing impairment and its impact on hearing loss deserves further studies.

## INTRODUCTION

Hearing plays an important role in the development of human beings. The peripheral hearing system is fully formed at birth, while the central auditory system takes two years to mature. This period corresponds to that of the greatest neuronal plasticity of the auditory pathway, and this maturing process depends on the quantity and quality of the external stimuli captured. Early identification and rehabilitation in cases of hearing loss are paramount for speech development (critical period and optimum period)^1^ and that of other cognitive functions at school age^2^.

Hearing acuity loss (hypoacusis) evolves silently, especially in the first years of life, when the child still does not complain of hearing loss. During childhood, it has a world prevalence of 1/1000 live births^3^^,^^4^, being higher (2–4%) among those coming from neonatal intensive care units (ICU)^2^^,^^5^. It is associated with genetic and environmental factors; however, in a significant number of children the etiology is unknown^4^^,^^6^. Among risk factors for hypoacusis, we have a family history of hearing deficit, craniofacial malformation, genetic syndrome, birth weight below 1000g, asphyxia, hyperbilirubinemia and mechanical ventilation use^5^.

Hearing losses may be classified according to type (conductive or sensorineural), laterality, symmetry, clinical characteristic (syndromic or not), time of onset (congenital, perinatal or postnatal), hereditary (genetic or not), time of manifestation (prelingual, peri-lingual or post-lingual)^4^. They may also be classified as to intensity in mild, moderate, severe and profound. In conductive hearing loss there is sound conduction impairment from the outer medium to the cochlea; and in sensorineural hearing loss there is cochlea and/or cochlear nerve (central auditory pathway) involvement.

It is well established the association between some congenital infections and hearing loss. The most frequent infectious agents associated with hearing loss are Cytomegalovirus (CMV)^7^, rubella virus^3^^,^^8^, ^9^, ^10^, Toxoplasma gondii and the herpes virus^11^. Recently, newborn with HIV are also being included in this risk group^12^. Children with congenital rubella have hearing deficit in up to 20% of the cases^4^^,^^9^ and those infected by CMV, especially the symptomatic ones, may have it in up to 40% of the cases.

Toxoplasma gondii has been associated with disorders of the auditory pathways since the 1950's^13^, when calcium deposits (similar to those found in the brains of children with congenital toxoplasmosis) were found in the spiral ligament and the cochlea^14^. Hearing loss has been reported in about 20% of the congenital toxoplasmosis cases, especially in untreated children or those treated for a very short period^4^^,^^15^^,^^16^. Eichenwald (1947), studied children who had not been treated in the first year of life and found hearing loss in 17% (12/70) of those children who had the neurologic form of the disease and in 10% (3/31) of those with the generalized form. Severe hearing loss has been almost exclusive of those cases bearing intense clinical manifestations.^17^; however, Wilson et al.^18^^,^^19^ reported unilateral or bilateral hearing loss in 5 (26%) of the 19 children with subclinical infection. Other authors have found no association between the parasites and hypoacusis, when children are treated^15^^,^^20^, and there still are doubts as to the rate at which toxoplasmosis can cause hearing deficit^13^^,^^17^^,^^21^.

Considering the little information available about significant hearing loss in children with congenital toxoplasmosis, especially those with subclinical infection, this study aims at characterizing the hearing loss pattern in children with congenital toxoplasmosis, identified at the neonatal screening process in Belo Horizonte, Minas Gerais.

## MATERIALS AND METHODS

From September, 2003 to October, 2004, we carried out a pilot project in the city of Belo Horizonte, Minas Gerais, for neonatal screening, using the IgM anti-T. gondii immune assay in dry blood, in order to assess the incidence of congenital toxoplasmosis in live births in the city and neonatal screening as a strategy for early diagnosis and treatment of infected children. According to 2004 data from SINASC/SES, the Neonatal Screening Program tested about 97% of newborns. The criteria used to define congenital toxoplasmosis were those accepted by a consensus on the theme: positive IgM and/or IgA in the first six months, or persistently positive IgG at 12 months of life^22^.

The infected children were treated (sulfadiazine, pyrimethamine and folinic acid) during 12 months^23^, followed for 24–36 months and submitted to multiprofessional evaluation which consisted of pediatric, ophthalmologic and auditory exams. Hearing assessment was carried out at the time of diagnosis and 12 months afterwards, by the Audiology Service of the UFMG-Medical School Hospital, based on Behavioral Audiometry, Transient and Distortion Product Evoked Otoacoustic Emissions, Immitanciometry and Brainstem Evoked Auditory Potential (BEAP). When necessary and with the informed consent of the child's guardian, BEAP was carried out under sedation in the Surgical Theater of the UFMG University Hospital (UFMG-UH). Should doubtful results be attained, the exams were repeated at different time points throughout the child's growth. Data collection for this study was interrupted when the child came to 24–36 months of life; however their follow up continued at the Departments of Pediatric Infectology and Audiology at the UFMG-UH. Those children with hearing impairment were referred to the speech and hearing therapy and hearing aid fitting when that was the case.

For speech and hearing anamneses, we used the UFMG-HU Department of Audiology protocol, which investigates the variables most commonly associated with hearing loss4: family history of hearing loss; gestational problems (infections of the TORCHS group, use of abortive and ototoxic drugs); delivery complications (hypoxia - Apgar between 0–4 in the 1st minute and 0–6 in the 5th minute, prematurity, low birth weight, hyperbilirubinemia, delivery trauma, ototoxic drugs, noise), post-natal complications (phototherapy for more than two days, incubator stay for more than five days, mechanical ventilation use for more than five days, repetition otitis, measles, bacterial meningitis, mumps, encephalitis, ototoxic drug use for more than five days, head injury, acoustic trauma, sepsis). We used a careful history taking as criteria to establish the possible etiologic associations, because it is considered the most efficient method^6^.

We used the following equipment in order to carry out the audiologic exams: (PA5) Interacoustics Pediatric Audiometer and Impedanciometer, ANSI S3.6/ISO 389 calibration pattern. In behavioral audiometry we used Brazilian musical percussion instruments (agogô, sino, guizo, reco-reco, coco, chocalho) which were standardized for the evaluation. Otoacoustic emissions were captured using Biologic's AuDX Plus device (protocols described on Table 1). BEAP was carried out with Biologic's Navigator device and the EP317 software. This piece of equipment has two recording channels. Responses were captured by means of silver electrodes placed on the ear lobes (A1 and A2), nose (Nz) and forehead (Fz), keeping the impedance below^5^ Kohms.

**Table 1:** Brainstem auditory evoked potentials recording characteristics.

STIMULUS	
Transducer	Supra-aural phones
Type	Click
Duration	0.1 msec
Velocity	13.1/s
Polarity	Rarefaction
Initial Power	85 dBHL
Ear	Monoaural
AQUISITION	
Gain (amplification)	100,000
Analysis time	15 msec
High-pass filter	3000Hz
Low-pass filter	30Hz
Number of scans	2 (1024 X2)
Contralateral masking	60dB (WN)

The normality criteria used in the auditory potential analysis was proposed by Gorga et al.^24^, which protocol follows the same criteria used in the tests carried out at the UFMG-UH Audiology ward.

For OAE recording, a probe was placed at the entrance of the external auditory meatus (EAM) which has a stimuli generator inside. As they hit the cochlea, these stimuli produce an echo - the otoemissions - which trails the inverse sound pathway, that is, from the inner ear to the middle and then to the external acoustic meatus, and then captured by a microphone placed in the EAM. If present, otoemissions hint to middle and external ear integrity6. Transient evoked otoacoustic emissions were considered present when their reproducibility was above 70% and the response amplitude / noise ratio (R/N) was above that or equal to 6 dB. Distortion product evoked otoacoustic emissions were considered present whenever the R/N ratio was higher than or equal to 6 dB.

The hearing loss variables investigated were: type (sensorineural or conductive), intensity (mild, moderate, severe and profound), laterality and symmetry.

In behavioral audiometry, the response patterns suggesting central auditory alterations were: exacerbated response, response latency increase, difficulty of localization with normal acuity, no habituation at repetitive stimuli, and no cochleo-eyelid reflex with normal hearing^25^.

Stimuli were psychoacoustically calibrated in dBHL (decibels pertaining to the 0 value of the normal young population obtained psychoacoustically). In order to classify hearing loss levels, we considered the guidelines from the Bureau International D'Audiophonologie (BIAP) which classifies the losses in mild (minimum response levels between 21–40 dBHL), moderate (minimum response levels between 41–70 dBHL), severe (minimum response levels between 71–90 dBHL) and profound (no response to the stimuli employed at a maximum output of 85 dBHL)^6^.

Responses were considered symmetrical when the hearing losses were classified at the same level of impairment on both sides. Asymmetry was of level 1 when the one side hearing loss classification was immediately adjacent in severity level to that of the contralateral side. The asymmetry was deemed of 2 levels when the classification of sides was not adjacent in levels of severity.

In order to build the database, we used the Epi Info version 6.0 statistical package. For analysis we also used the StatXact software to perform Fisher's Exact Test. We used the Student t test in order to compare the mean values and the Fisher's Exact Test to compare gender and risk factor distribution. This study was approved by the COEP-UFMG (ETIC 157/01) and we honored the standards established for research with human beings, according to resolution 196/96 from CONEP.

## RESULTS

From September 2003 to October 2004, 30,808 children (about 97% of live births) were screened in Belo Horizonte (BH) and 20 were identified with congenital toxoplasmosis, resulting in a rate of 1:1590 infected by live births. In relation to the time of diagnosis for these 20 children, six (30%) had already been identified during prenatal care (prenatal screening program implemented in BH), four (20%) were identified in the neonatal period because they had clinical manifestations suggesting the infection; and ten (50%) were identified by neonatal screening alone.

Among these 20 children identified by neonatal screening in BH, one was taken off the analysis because for having died in the neonatal period. We carried out behavioral auditory evaluations and Otoacoustic Emission tests (EOAE) in 19 children and Brainstem Evoked Auditory Potential - BEAP in 17. We noticed that 13 children (68.4%) had normal evaluation for their ages, and six had hearing deficit - four (21.1%) were sensorineural and two (10.5%) had conductive hearing loss. There was no gender wise difference among the three groups (p=0.99). On Table 2 we list some of the risk factors for hypoacusis, as well as the frequency observed. According to Fisher's Exact Test, we did not detect any difference among the three groups as to any of the risk factors. One child had, besides toxoplasmosis, two other risk factors (birth weight below 1,500g and were under mechanical ventilation for one month), and however his hearing was deemed normal.

**Table 2:** Distribution of hearing loss risk factors in 19 children with congenital toxoplasmosis identified by the neonatal screening in Belo Horizonte from September 2003 to October 2004.

Hearing loss risk factors	Normal hearing n=13	Hearing loss n=6	
		Conductive n=2	Sensorineural n=4
Cranio-facial anomalies	0	1	0
Birth weight < 1,500g	1	0	0
Apgar between 0 – 4 minutes	1	0	0
Apgar between 0 – 6 minutes	1	0	0
Mechanical ventilation^3^ 5 days	1	1	1

In the population studied, we did not observe the following risk factors for hearing loss: family history, other TORCH intra-uterine infections, hyperbilirubinemia, use of ototoxic drugs, bacterial meningitis, parents who used drugs/alcohol, syndromes.

Table 3 shows the mean, median and standard deviation of the birth weight of the 13 children with normal audiometry and those from the six children with hearing deficit (four classified as sensorineural and two as conductive). The difference among the mean values of birth weight associated with the two groups did not show statistical significance (p=0.51).

**Table 3:** Birth weight mean, median and standard of the 19 children with congenital toxoplasmosis identified by neonatal screening in Belo Horizonte, from September 2003 to October 2004.

Statistics	Normal hearing (n=13)	Hearing loss (n=6)
Mean	2,612 g	2,803 g
Median	2,590 g	2,890 g
Standard deviation	611 g	494 g

As far as neonatal signs and symptoms are concerned the group without hearing alterations had three children with systemic manifestations (liver and spleen enlargement and/or petechias) and, of these, two also had hydrocephaly and microphthalmia. Among the children with hearing impairment we observed that one presented systemic manifestation and prematurity (Table 4). After more than 12 months of follow up, only one child from the first group and one from the second presented with neuropsychomotor development delay.

**Table 4:** Characteristics of children with congenital toxoplasmosis and sensorineural hearing loss according to neonatal screening in Belo Horizonte, from September 2003 to October 2004.

Code / DN	S	IG	PN (g)	Apgar 1' / 5'	TS	I (days)	MF	VM	Clinical manifestations	TT	Age of hearing assessment (in months		Sensorineural assessment result
											Behavioral and OAE	BEAP	
BXX1744 29/07/03	M	34	1970	9/9	41	S (35)	N	S (35)	Premature, low birth weight, systemic disease, bilateral macular retinochoroiditis.	12	7, 11	11	Severe right side deficiency and profound to on the left
BYP1585 15/08/04	M	38	2820	NS		N	N	N	bilateral macular retinochoroiditis, DNPM delay	3	7, 22	22	Mild left side retrocochlear deficiency
BXU1595 12/09/03	F	38	2960	8/7	N	N	N	N	Right side macular retinochoroiditis	12	2, 18, 24	2, 21, 26	Mild left side deficiency
BYA3384 22/12/03	F	38	2540	9/9	N	N	N	N	bilateral macular retinochoroiditis	10, irregular	2	2	Bilateral moderate deficiency

DN=date of birth; S=gender; IG=gestational age in weeks; PN=birth weight; TS=blood transfusion; I=incubator > 5 days; MF= facial malformation; VM=mechanical ventilation ^3^ 5 days; TT=treatment time in months; N=no; S=yes; NS=does not know; EOA=otoacoustic emissions; PEATE=Brainstem Evoked Auditory Potential

**Figure 1:**
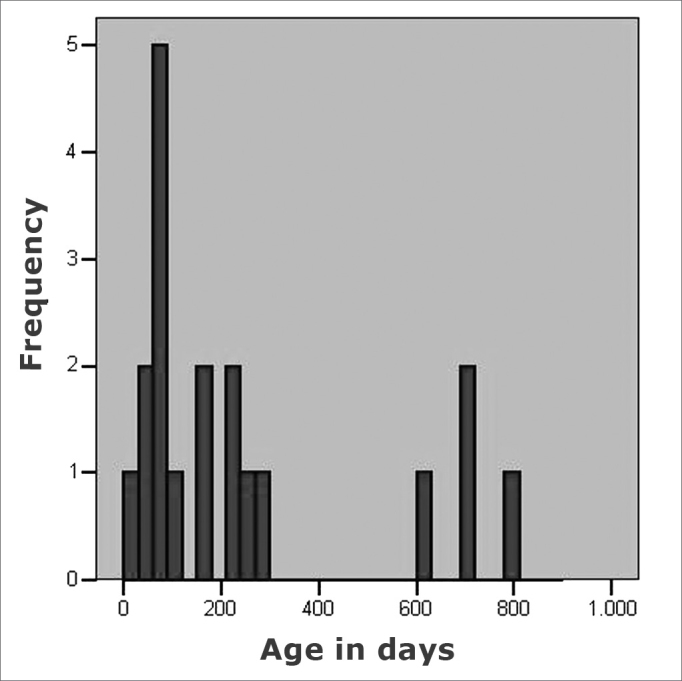
Age, in days, after the firs auditory evaluation in 19 children with congenital toxoplasmosis, identified by neonatal screening.

As to the age when the children were submitted to the first hearing evaluation, we observed a median value of 157 days, and 75% of the children were first assessed before 270 days. However, as we can see in Figure 1, four children underwent their first hearing assessment only at the end of their second year of life. The last hearing assessment was carried out at the end of their second year of life. The last hearing evaluation was carried out at the end of their second year of life (median value of 624 days), noticing that 75% of the children underwent such evaluation before 710 days of life.

Among the children with sensorineural deficit (n=4), the hearing loss was severe and profound in one, moderate in one and mild in two children; and was asymmetrical in only one of the children with bilateral involvement (Table 4). Only one of the children, the most severely affected one, had other risk factors for developing hypoacusis - she was under mechanical ventilation for about one month. This child presented clear signs and symptoms of congenital toxoplasmosis: prematurity, liver and spleen enlargement, low birth weight (1,970g) and bilateral retinochoroiditis. The other three children, one with moderate bilateral deficit and two with mild unilateral deficit, were born asymptomatic and were identified only by neonatal screening. Two of these children were referred to wear hearing aids and undergo speech and hearing therapy. The children with conductive hearing loss were referred to otolaryngological evaluation. Two children, in whom it was possible to provide proper treatment with anti-parasitic drugs, continued to present hearing deficit, contrasting with the findings from other authors.

In three children without hearing impairment, who underwent BEAP evaluation in the first three months of life, and repeated the test after 18 months, we noticed hearing deficit in the first exam and a normal results in the second, suggesting a slower neural maturation in these children.

In four children deemed normal by the BEAP at two years of age, in the first hearing evaluation there were not TEOAE, and there were DPOAE, suggesting that these may be more sensitive to audiologic diagnosis.

## DISCUSSION

There is a consensus about the importance of early hearing deficit diagnosis for a better development of language, cognition and child socialization. Neonatal hearing screening, recently mandatory as public health policy, will slowly reach the universe of newborns, however, risk factors identification for hearing loss allows us to follow children up and to reassess them at the end of the first year of life, thus increasing the auditory screening sensitivity. Therefore, there is the need to implement effective strategies to identify risk factors associated with this deficiency. Among the risk factors, toxoplasmosis - a prevalent disease in Brazil, has not been often associated with hearing deficits. However, identifying children with sensorineural hearing loss (4/19), among those screened with congenital toxoplasmosis, brings up this discussion about the impact of this congenital infection in children hearing deficiencies.

Among the 20 children identified by neonatal screening, 15 (75%) have subclinical infection and, of these, nine were identified by neonatal screening only.

Recent epidemiological studies^16^^,^^21^ aiming at associating toxoplasmosis and hypoacusis observed a higher risk of hearing deficit among children who were positive for T. gondii; however, these studies are not prospective, and for this reason are unable to assess other risk factors for the aforementioned deficit. In the present study it was possible to confirm the diagnosis of congenital toxoplasmosis, rule out other infectious agents associated with congenital infections and, during follow up of these children, to rule out other risk factors associated with such deficit (meninges infections, middle ear and external ear infections). Among the four (21.1%) children identified with sensorineural hearing loss, one presented other risk factors for the deficit, two presented mild unilateral deficit with little functional repercussion and one presented moderate bilateral deficit. The last three children were born asymptomatic and identified only by neonatal screening and did not show any other reason, except toxoplasmosis, to explain the findings, showing that such parasitosis, even in the absence of other clinical manifestations, must be considered in the assessment of children with hearing loss.

Longitudinal studies with newborns followed up during long periods pointed to a high index of abstention from the mothers at scheduled return visits^2^. In four cases of our study, the medical visits did not occur at the required regular intervals; however, all the children came for at least one hearing evaluation, and this may be associated to the fact that the guardians knew that the child had some risk factor for hearing loss. The delay seen in four children to undergo the first hearing assessment may be attributed to the work or maternal disease, mother's lack of interest, difficulty in transporting the child to the audiology ward, difficulty in handling the child for exam purposes, and others. Efforts must be made in order to identify early on the prevalent congenital infections, including toxoplasmosis, facilitating early access to audiology services.

Long standing treatments (one year) for congenital toxoplasmosis have been associated to better children prognosis by some authors^15^^,^^20^. Among the four children in the present study, one received antiparasitic drugs for three months only (this mother, a teenager, was not interested in her child's problem) and the other was irregularly treated for 10 months (did not regularly come for visits at the ward); the most affected child and the other one with unilateral hearing loss were treated during 12 months.

Table 5 shows comparisons of three studies which encompassed hearing evaluation in children with congenital toxoplasmosis. The Chicago Study^15^^,^^20^ evaluated children treated with the classical antiparasitic drugs for 12 months; Wilson et al's study.^19^ describes the findings from 19 children with subclinical infection who did not receive specific treatment, or received it for a very short time; and the present study, in which among the 19 children treated three were not properly treated (little time) and, of these, two had hearing deficits.

**Table 5:** Results from auditory evaluation in children with congenital toxoplasmosis submitted to treatment or not, during the first year of life.

Definitions					Results of the sensorineural auditory evaluation		
Hearing loss level	Chicago's collaborative studya		Wilson et al.^b^	Present investigation	Chicago's studya	Wilson et al. bsubclínica	Present investigation
	BERA (dB/HL)	Audiogram (dB/HL)					
Normal	≤ 20	0–20	< 25 dB	≤ 20	55	14	15
Mild	> 20–40	25–40	25–50	> 20–40	0	3 (15,8%)	2 (10,5%)
Moderate	> 40–60	>40	51–80	> 40–70	0	2 (10,5%)	1 (5,3%)
Severe	>60	> 70–90	NE	>70–90	0	0	1 (5,3%)
Profound		>90	NE	> 90	0	0	
Total					55	19	19

NE=not found; BERA=cerebral auditory response; dB=decibels; HL=hearing level; NA=not assessed; Adapted from McGee et al.^15^ a- Children treated in the first year of life, during 12 months^15^^,^^20^^,^^28^ b- Children, asymptomatic at birth and not treated or treated for a very short period, one month^19^

The results observed in this study are similar to those found by Wilson et al.^19^ Studies with larger samples, submitted to long stand treatment (12 months), did not identify the presence of hypoacusis, concluding for a better prognosis of the treated children^20^. In the present investigation, among the 16 children properly treated, for long time and regularly, two presented hearing deficit, but only one had an important functional loss. This child was born severely affected by the infection and presented other risk factors for hearing loss besides parasitosis (low birth weight and long term use of assisted ventilation), and developed hearing and ocular sequelae despite treatment. Hearing loss occurring because of assisted ventilation for more than five days can be associated with the noise produced by the breathing devices, mechanical ventilation duration and involved pulmonary pathologies^5^. As far as birth weight is concerned, although such child presented with low birth weight, hypoacusis risk is greater in newborn with birth weight below 1,500g, although we can not disregard perinatal care situations^5^. Therefore, in this child we can not relate hearing loss with toxoplasmosis, although the clinical picture she had characterizes a severe disease stage.

As to hearing loss intensity and its functional meaning, studies have assessed the importance of unilateral or bilateral mild hearing losses for the development of children and have suggested that these losses may affect many domains such as language skills, speech perception, auditory functional skills, academic performance, socio-emotional development, motor skills including early movements and coordination^26^. In general, these losses have been identified later on, between 5–6 years of age^2^ and the knowledge regarding a risk factor for the loss, such as toxoplasmosis, may help speed up the access of such children to an audiology service or speech and hearing therapy.

A historical cohort study carried out at the Pediatric Infectology Service of the UFMG-UH, evaluating BEAP audiometry in 49 children with congenital toxoplasmosis, most of them with early manifestations of the infection, and followed for 10.3 years in average, showed alterations in five cases (10.2%) and no association between treatment type in the first year of life and hearing deficit^27^. In the present study, proper treatment of two of these children with hearing loss did not help avoid the loss, and this is different from the results attained by McGee et al.^15^ and McLeod et al.^20^ who reported hearing normality in treated children, even when they had neurologic and ocular involvement. The follow up of these children may provide new data to clarify these issues.

## CONCLUSION

The results observed in the present study suggest that congenital toxoplasmosis, common in Brazil, is a risk factor for hearing loss; and studies assessing the impact of the subclinical infection on hearing loss are imperative. Facing the possibility of implementing a prenatal or neonatal screening program for congenital toxoplasmosis, its planning must include early auditory evaluation for identified cases. Toxoplasmosis, especially when asymptomatic at birth, may be included among the “Unknown causes” of hearing loss and all effort should be put up in order to identify the children at risk and allow for early diagnosis and treatment, which have been associated with a significantly better prognosis^20^.
